# Supplementary material for: A UMLS-based spell checker for natural language processing in vaccine safety
Source: BMC Med Inform Decis Mak. 2007 Feb 12;7:3. doi: 10.1186/1472-6947-7-3 (PMC1805499; doi:10.1186/1472-6947-7-3)
Supplement: Additional file 13 — RAP application directory. Sets up directory for RAP (RDF application for PHP) source code [file 1472-6947-7-3-S13.gz › rap/doc/phpdoc/dataset/Dataset.html]

xml version="1.0" encoding="iso-8859-1"?


Docs For Class Dataset


## Class Dataset

Description

Description |
Descendents
| Methods (details)

Dataset implementation.

Superclass of datasetMem and datasetDb which contains shared functionality.

History:

- access: public
- author: Daniel Westphal (http://www.d-westphal.de)
- author: Chris Bizer <chris@bizer.de>
- version: V0.9

Located in /dataset/Dataset.php (line 21)

Direct descendents

Description |
Descendents
| Methods (details)

| Class | Description |
| --- | --- |
| DatasetDb | Dataset implementation. |
| DatasetMem | In-memory implementation of a RDF dataset. |

Method Summary

Description |
Descendents |
Methods (details)

void
loadFromFile
(string $file)

void
loadFromString
(string $string)

void
serializeToFile
(string $fileName)

string
serializeToString
()

Methods

Description |
Descendents |
Methods (details)

loadFromFile (line 29)

Load a Dataset from File

- access: public

void

loadFromFile
(string $file)

- string
  $file

loadFromString (line 41)

Load a Datset from a string

- access: public

void

loadFromString
(string $string)

- string
  $string

serializeToFile (line 53)

Serialize the Dataset to File

- access: public

void

serializeToFile
(string $fileName)

- string
  $fileName

serializeToString (line 65)

Serialize the Dataset to string

- access: public

string

serializeToString
()

Documentation generated on Thu, 7 Jul 2005 13:41:09 +0200 by phpDocumentor 1.3.0RC3
